# Type I interferon signaling deficiency results in dysregulated innate immune responses to SARS‐CoV‐2 in mice

**DOI:** 10.1002/eji.202249913

**Published:** 2022-09-15

**Authors:** Patricia P. Ogger, Minerva Garcia Martín, Christina Michalaki, Jie Zhou, Jonathan C. Brown, Yue Du, Kamran M. Miah, Omar Habib, Stephen C. Hyde, Deborah R. Gill, Wendy S. Barclay, Cecilia Johansson

**Affiliations:** ^1^ Section of Respiratory Infections, National Heart and Lung Institute Imperial College London, St Mary's Campus; ^2^ Department of Infectious Disease Imperial College London, St Mary's Campus; ^3^ Radcliffe Department of Medicine (NDCLS) University of Oxford

**Keywords:** Innate Immune Response/ type I IFN, In vivo, SARS‐CoV‐2/ myeloid cells

## Abstract

SARS‐CoV‐2 is a newly emerged coronavirus, causing the global pandemic of respiratory coronavirus disease (COVID‐19). The type I interferon (IFN) pathway is of particular importance for anti‐viral defence and recent studies identified that type I IFNs drive early inflammatory responses to SARS‐CoV‐2. Here, we use a mouse model of SARS‐CoV‐2 infection, facilitating viral entry by intranasal recombinant Adeno‐Associated Virus (rAAV) transduction of *hACE2* in wildtype (WT) and type I IFN‐signalling‐deficient (*Ifnar1^–/–^)* mice, to study type I IFN signalling deficiency and innate immune responses during SARS‐CoV‐2 infection. Our data show that type I IFN signaling is essential for inducing anti‐viral effector responses to SARS‐CoV‐2, control of virus replication and to prevent enhanced disease. Furthermore, *hACE2‐Ifnar1^–/–^* mice had increased gene expression of the chemokine *Cxcl1* and airway infiltration of neutrophils as well as a reduced and delayed production of monocyte‐recruiting chemokine CCL2. *hACE2*‐*Ifnar1^–/‐^* mice showed altered recruitment of inflammatory myeloid cells to the lung upon SARS‐CoV‐2 infection, with a shift from Ly6C^+^ to Ly6C^–^ expressing cells. Together, our findings suggest that type I IFN deficiency results in a dysregulated innate immune response to SARS‐CoV‐2 infection.

This article is protected by copyright. All rights reserved

### Keywords:

Innate Immune Response/ type I IFN / In vivo / SARS-CoV-2/ myeloid cells

For Peer Review

### Introduction

The ongoing coronavirus disease (COVID-19) pandemic caused by severe acute respiratory syndrome-coronavirus 2 (SARS-CoV-2) has resulted in over 400 million cases in the first two years of the pandemic. The estimated fatality rate lies between 1-2%, however this is considerably higher for elderly patients over 80 years of age (~10%) and nursing home residents (>20%) (1). Type I interferons (IFNs) are one of the first responses elicited against viral infection and they induce anti-viral defense mechanisms by binding to the IFN-α/β receptor (IFNAR) and signaling through the JAK-STAT pathway. This induces expression of IFN stimulated genes (ISG), resulting in expression of anti-viral effector proteins that restrict viral replication and activation of immune cells via induction of chemokine and cytokine production (2), including CXCL10 and CCL2 (3). Early *in vitro* studies using human bronchial epithelial cell lines infected with SARS-CoV-2 showed decreased production of type I and III IFNs coupled with low anti-viral defense signals and a pro-inflammatory environment compared to infection with influenza A virus (IAV) (4). Furthermore, in severe and critically ill COVID-19 patients, an impaired type I IFN response has been observed, resulting in decreased viral clearance (5). A lack of an efficient type I IFN response in these patients is in part due to inborn errors of type I IFN immunity (6) or circulating auto-antibodies neutralizing type I IFNs (7). Also, a recent animal study has identified that type I IFN signaling is required for the recruitment of pro-inflammatory cells into the lungs following SARS-CoV-2 infection (8). These findings highlight the importance of functional type I IFN responses for anti-viral defenses against SARS-CoV-2.

Using a mouse model of SARS-CoV-2 infection, facilitated by intranasal recombinant Adeno-Associated Virus (rAAV) induced expression of human angiotensin converting enzyme 2 (hACE2), this study investigated the dynamics of innate immune responses to infection with SARS-CoV-2 in the context of type I IFN signaling impairment. Overall, the data show that type I IFN signaling is essential to induce anti-viral responses and control viral replication and disease severity during SARS-CoV-2 infection. Furthermore, type I IFN signaling-deficient mice show dysregulated innate immune responses to SARS-CoV-2 infection, marked by increased neutrophil recruitment into the airways and delayed recruitment of myeloid inflammatory cells.

### Results and discussion

To investigate the dynamics of innate, anti-viral immune responses to SARS-CoV-2 infection in the context of type I IFN-signaling deficiency, 8–12-week-old C57BL/6 wildtype (WT) or *interferon alpha receptor-1*$^{-/-}$ (*Ifnar1*$^{-/-}$) mice were intranasally transduced with rAAV9 containing either *hACE2* or *eGFP* (control) as published recently (9), followed by intranasal infection with 2x$10^6$ PFU SARS-CoV-2

For Peer Review

(D614G, first wave isolate) 20 days later and study endpoints were at 2, 4 and 8 days post infection (d.p.i.) (Figure 1A). Gene expression analysis of *hACE2* in lung tissue 20 days post administration of rAAV9 (before infection) showed similar expression in WT and *Ifnar1*$^{-/-}$ mice relative to *Gapdh* (Figure 1B). Cryo-sectioning of lung tissue 20 days post administration of rAAV9-*eGFP* (before infection) furthermore showed similar distribution of *eGFP* in WT and *Ifnar1*$^{-/-}$ mice (Supp. Figure 1A), suggesting similar rAAV transduction efficacy in both groups of mice. Upon infection with SARS-CoV-2, *hACE2-Ifnar1*$^{-/-}$ mice showed increased weight loss, peaking at 6 d.p.i., compared with *hACE2*-WT mice (Figure 1C). Furthermore, plaque assays on Vero cells overexpressing SARS-CoV-2 binding receptors ACE2 and transmembrane protease serine 2 precursor TMPRSS2 (VAT cells) showed that IFNAR1-deficiency results in significantly higher viral loads at day 2 and 4 post infection with SARS-CoV-2 compared to *hACE2*-WT mice (Figure 1D). Gene expression of SARS-CoV-2 nucleocapsid (N) and envelope (E) genes was significantly higher in *hACE2-Ifnar1*$^{-/-}$ mice compared with *hACE2*-WT mice from 2 d.p.i. (Figure 1E and Supp. Figure 2B). Since AAV-e*GFP* transduced *Ifnar1*$^{-/-}$ mice did not become infected with SARS-CoV-2 and did overall not differ significantly from AAV-e*GFP* transduced WT mice (Supp. Figure 2), this group was not included in all experiments to reduce animal numbers. Together, these data show increased viral load in *hACE2* expressing IFNAR1-deficient mice upon SARS-CoV-2 infection, measured both by plaque assay and N and E gene expression. Previous studies have shown similar trends (8), although differences between WT and *Ifnar1*$^{-/-}$ are more pronounced in the model used here. This could be due to several factors: here, Vero cells overexpressing hACE2 and TMPRSS2 were used for plaque assays, allowing for better viral replication, while the isolate used for infection was hCoV-19/England/IC19/2020, which harbors the D614G spike mutation as opposed to USA-WA1/2020, enhancing viral replication (10). Together, these factors might explain why we detected higher viral loads and more prominent differences emerged between *hACE2*-WT and *hACE2-Ifnar1*$^{-/-}$ mice.

To investigate the impact of impaired type I IFN signaling during SARS-CoV-2 infection, we first assessed type I IFN expression upon infection. At 2 d.p.i. both IFN-α and IFN-β were significantly increased in BAL fluid of *hACE2*-WT compared to e*GFP*-WT mice (Supp. Figure 1B). In the *hACE2-Ifnar1*$^{-/-}$ group, expression of IFN-α was significantly lower, while IFN-β levels were higher compared to the *hACE2*-WT group. We next investigated ISG expression after SARS-CoV-2 infection, since in *Ifnar1*$^{-/-}$ mice limited amounts of type I IFN cytokines can be produced but cannot signal for downstream ISGs induction. Chemokine *Cxcl10* and anti-viral effectors *Mx1, Oas1* and *Viperin* were quantified. The expression of these ISGs was increased in *hACE2*-WT mice upon infection with SARS-CoV-2 at 2 d.p.i., however in *hACE2-Ifnar1*$^{-/-}$ mice, ISG expression was significantly reduced, but not completely absent (Figure 1F and Supp. Figure 3 C-F). These results suggest an initial increase of IFN-β in *hACE2-Ifnar1*$^{-/-}$ mice, which may be due to higher viral titers, but that is not translated into ISG expression due to the

For Peer Review lack of signaling through the IFNAR1. We therefore investigated the expression of type III IFNs, IFN-λ2-3, which can contribute to ISG expression. IFN-λ expression was induced upon infection in *hACE2*-WT mice at 2 d.p.i. while remained at baseline levels in the *hACE2-Ifnar1*$^{-/-}$ mice (Supp. Figure 1C). Furthermore, *Ifng* gene expression was significantly increased in *hACE2- Ifnar1*$^{-/-}$ mice later during the infection, by 4 d.p.i. (Supp. Figure 4A). This correlated with CD3$^{+}$ T cell recruitment to the airways (Supp. Figure 4B-D). Our data suggest that limited levels of type I or III IFNs are produced early during infection in the *Ifnar1*$^{-/-}$ mice resulting in some ISG expression but overall, these data suggest that type I IFN signaling is the main driver for inducing cell intrinsic anti-viral responses.

We next assessed the gene expression of inflammatory mediators and found that expression of the chemokine *Cxcl1*, which is not dependent on type I IFN signaling (3), was increased in *hACE2-Ifnar1*$^{-/-}$ mice at 2 and 4 d.p.i. compared to *hACE2*-WT (Figure 2A and Supp. Figure 3G). As CXCL1 plays an essential role in early host immune responses by recruiting neutrophils (11), we next analysed infiltration of neutrophils (gated as live, CD45$^{+}$, Ly6G$^{+}$, Supp. Figure 5A) into the airways at 2 d.p.i. In line with highly increased gene expression of *Cxcl1*, neutrophil recruitment to the airways (BAL) was significantly increased in *hACE2-Ifnar1*$^{-/-}$ mice at 2 d.p.i., both proportional of leukocytes (CD45$^{+}$ cells) and in total numbers (Figure 2B and C). This was recapitulated in lung tissue with increased proportions of neutrophils in type I IFN signaling-impaired mice at 2 d.p.i. (Figure 2D and Supp. Figure 3H), decreasing over time. Taken together, these findings suggest that during SARS-CoV-2 infection, type I IFN signaling deficiency results in increased neutrophil recruitment via CXCL1, thereby contributing to a pro-inflammatory environment. Indeed, *Cxcl1* is also increased in *Ifnar1*$^{-/-}$ mice during influenza A with secondary pneumococcal infection (12), but decreased during RSV infection in mice (13), highlighting a pathogen specific CXCL1 response. Furthermore, since we show similar trends for viral load and neutrophil recruitment upon SARS-CoV-2 infection (both significantly increased in *hACE2-Ifnar1*$^{-/-}$ mice), which is a mechanism present in other respiratory viral infections such as respiratory syncytial virus (RSV) (13, 14), it will be important to further investigate the link between neutrophil recruitment and viral load in this model.

Since monocyte recruitment to the airways and lungs is key to early host responses to viral infection, we next investigated the expression of monocyte recruiting chemokine CCL2 and the recruitment of inflammatory myeloid cells. CCL2 protein expression was increased in BAL fluid of *hACE2*-WT mice at 2 d.p.i. with SARS-CoV-2 (Supp. Figure 3I). However, in *hACE2-Ifnar1*$^{-/-}$ mice CCL2 expression was significantly lower at 2 d.p.i., peaking at 4 d.p.i. at lower levels than in IFNAR1-sufficient mice (Figure 3A). These findings are in line with a report identifying early CCR2 signaling essential to restrict viral

For Peer Review burden in the lung in a mouse model of SARS-CoV-2 infection (15). The recruitment of CD64$^+$CD11b$^+$ inflammatory myeloid cells to the lung followed similar kinetics, as in *hACE2*-WT mice proportions were highest at 2 d.p.i. and subsequently decreased, while in IFNAR1-deficient mice proportions and total numbers of CD64$^+$CD11b$^+$ inflammatory myeloid cells strongly increased between 2 and 4 d.p.i. and were highest at 8 d.p.i. (Figure 3B and Supp. Figure 3J). We next assessed expression of the monocyte/macrophage differentiation antigen Ly6C within this population, since previous studies reported the infiltration of CD64$^+$CD11b$^+$Ly6C$^+$ inflammatory myeloid cells into the lung during SARS-CoV-2 infection (8, 15). This showed highly increased proportions of CD64$^+$CD11b$^+$Ly6C$^+$ in *hACE2*-WT but not IFNAR1-deficient mice at 2 d.p.i. in the BAL (Supp. Figure 5D) and lung (Figure 3C-D and Supp. Figure 3K), suggesting type I IFN dependency for recruitment. However, as we have previously shown that Ly6C is gradually downregulated on monocytes during response to respiratory viral infection (16), we also analyzed CD64$^+$CD11b$^+$ Ly6C$^-$ cells. The presence of CD64$^+$CD11b$^+$ Ly6C$^-$ inflammatory myeloid cells in the airways was not type I IFN signaling dependent, since both proportions and total numbers were significantly increased in *hACE2-Ifnar1*$^{-/-}$ mice at 4 and 8 d.p.i. (Figure 3C and E), while at 2 d.p.i. in the airways no significant differences emerged (Supp. Figure 5F). This accounts for the delayed emergence of inflammatory myeloid cells in the lung during type I IFN signaling impairment shown in Figure 3B and overall indicates altered recruitment dynamics of inflammatory myeloid cells. Taking these data together, our model recapitulates the deficiency of type I interferon responses seen in severe SARS-CoV-2 infection, which in patients is marked by decreased IFN-α, type I IFN activity and ISG score, as well as neutrophilia and increased CCL2 (5). Our data suggest that the lack of type I IFN signaling results in dysregulated innate immune responses in the lung during SAR-CoV-2 infection.

### Concluding Remarks

In summary, using a mouse model of SARS-CoV-2 infection we show that type I IFN signaling is essential for inducing anti-viral effector responses, control of virus replication and disease severity. Our data indicate that type I IFN signaling-deficient mice express increased levels of *Cxcl1* in the lung and increased infiltration of neutrophils to the airways compared to WT controls. Furthermore, we found reduced and delayed production of CCL2 and altered recruitment of inflammatory myeloid cells during IFNAR1-deficiency. This, together with an increased viral burden is associated with more severe disease in type I IFN signaling-deficient mice. The data shown here will be valuable for better understanding how impaired type I IFN signaling drives SARS-CoV-2 pathology and disease severity, which is highly relevant considering the large contribution of impaired type I IFN responses on life-threatening SARS-CoV-2 infections (6, 7) and deaths (17) and for the development of type I IFN-based treatment options for COVID-19 in vulnerable populations. To conclude, our findings show that type I

For Peer Review

IFN deficiency results in dysregulated innate immune responses to SARS-CoV-2 infection in the rAAV-*hACE2* mouse model.

## Materials and Methods

### Mice

C57BL/6 mice were purchased from Charles River UK Inc. *Ifnar1*$^{-/-}$ mice on a C57BL/6 background were bred in-house. All mice were bred and maintained in pathogen-free conditions and 8–12-week-old mice were used for experiments. All animal experiments were reviewed and approved by the Animal Welfare and Ethical Review Board (AWERB) at Imperial College London and approved by the UK Home Office in accordance with the Animals Act 1986 (Scientific Procedures) and ARRIVE guidelines. Both male and female mice were used for experiments after excluding sex bias in preliminary experiments. All experiments were performed twice, independently, per time point.

### rAAV vector production

The production, purification, and titration of rAAV2/9-*eGFP* or *hACE2* vectors were performed as previously described (9). Briefly, the respective rAAV vector was produced by polyethylenimine (PEI, PolySciences)-based triple transfection of human embryonic kidney (HEK) 293T/17 cells (ATCC, CRL-11268). The AAV plasmids transfected included the Adenovirus helper plasmid (pAdDeltaF6), AAV Rep-Cap pAAV2/9 plasmid and the transgene plasmid. The transgene plasmid containing *eGFP* or *hACE2* was engineered to include a lung-optimized hCEFI (human Cytomegalovirus enhancer/elongation factor 1 alpha) promoter (18), Woodchuck Hepatitis Virus Post-transcriptional Regulatory Element (WPRE) (19) and mir142-3pT (20). rAAV particles were concentrated and formulated into PBS using 100 kDa Ultra centrifugal filters (Amicon, Merck) after iodixanol gradient centrifugation. Physical titre (DNase-resistant genome copies, DRGC/mL) was determined by quantitative polymerase chain reaction (qPCR) analysis with primers and a probe against WPRE (21). Purity of vectors was confirmed by analyzing 20 µl of diluted vector on 4-12% SDS polyacrylamide gels, where total protein was visualized using Coomassie stain according to the manufacturer's protocols (Life Technologies).

### hACE2 transduction

For transduction, WT or *Ifnar1*$^{-/-}$ mice were lightly anesthetized and instilled i.n. with $1x10^{11}$ DNase Resistant Gene Copies (DRGC) rAAV9-*eGFP* or rAAV9-*hACE2* in 100 µl PBS. *hACE2* gene expression in whole lung homogenate was assessed at day 20 post instillation by relative quantification to *Gapdh* using primers and probes for *hACE2* listed in the key resource table.

For Peer Review

**Cryosectioning and native e*GFP* detection**

Mice were sacrificed 20 days post instillation of AAV-e*GFP* or PBS and lungs were removed after inflation with 4% PFA. After 24-hour fixation in 4% PFA, lungs were inflated with 30% sucrose and submerged in 30% sucrose for 24 hours. Lungs were subsequently inflated with 1:1 cryo embedding matrix (OCT)/30% sucrose and individual lobes were submerged in OCT/30% sucrose in plastic molds and frozen at -80 °C. Left lungs were cryosectioned to produce 7 µm thick sections, mounted using DAPI-supplemented mounting media with coverslip, and e*GFP* expression was detected by fluorescent microscopy using the EVOS FL Auto 2 system (Thermo Scientific).

**Virus and infections**

First wave SARS-CoV-2 (D614G, isolate of hCoV-19/England/IC19/2020) was grown in African green monkey kidney cells overexpressing human ACE2 and TMPRSS2 (Vero-ACE2-TMPRSS2; VAT cells) (22). For infection 20 days post transduction with rAAVs, mice were lightly anesthetized and instilled i.n. with $2x10^6$ plaque forming units (PFU) of SARS-CoV-2 in 100 µl volume. SARS-CoV-2 titre was assessed in lungs at 2, 4 and 8 d.p.i. using a plaque assay. In brief, serial dilutions of lung homogenate in serum-free Dulbecco's Modified Eagle Medium (DMEM, containing 1% non-essential amnio acids (NEAA), 100U/ml Penicillin and 100 µg/ml Streptomycin) were performed and inoculated onto VAT cells for 1 h at 37°C. The inoculum was then removed and replaced with overlay medium (1x MEM, 0.2% w/v BSA, 0.16% w/v $NaHCO_3$, 10 mM HEPES, 2 mM L-Glutamine, 100 U/ml penicillin, 100 µg/ml streptomycin and 0.84% agarose). Plates were incubated for 3 days at 37°C before overlay was removed and cells were stained for 1 h at room temperature in 2x crystal violet solution. Virus plaques were counted and multiplied by the dilution factor to calculate titer as PFU/ml.

**Isolation of lung cells**

Mice were sacrificed at 0.75, 2, 4 and 8 d.p.i. and lungs were perfused with PBS. To obtain lung leukocytes, lung lobes were cut into smaller pieces and incubated in complete DMEM (cDMEM, supplemented with 10% fetal bovine serum, 2mM L-glutamine, 100 U/ml penicillin and 100 µg/ml streptomycin), 1mg/ml Collagenase D (Roche) and 30 µg/ml DNase I (Invitrogen) for 1h at 37°C and then mashed through a 100-µm filter (BD). Red blood cells were lysed using Ammonium-Chloride-Potassium buffer.

**BAL cell processing**

BAL was collected by flushing the lungs three times with 1 ml PBS supplemented with 5 mM EDTA (Life Technologies). BAL cells and supernatant were separated by centrifugation and BAL supernatants

For Peer Review were exposed to UV light for 2 min to inactivate SARS-CoV-2. Red blood cells were lysed using Ammonium-Chloride-Potassium buffer.

**Flow cytometry**

After red blood cell lysis, lung and BAL cells were incubated for 30 min with fixable live-dead Aqua dye (Invitrogen), followed by fixation for 30 minutes with 4% paraformaldehyde (PFA) to inactivate virus. Cells were then incubated for 20 min with a purified rat $IgG_{2b}$ anti-mouse CD16/CD32 receptor antibody (BD) to block Fc binding, followed by staining with fluorochrome-conjugated antibodies against CD45 (30-F11, BV605), CD26 (H194-112, BV711), Siglec-F (E50-2440, BV786), Ly6G (1A8, AF488), Ly6C (12HK1.4, PE), CD11c (HL3, PE-CF594), CD64 (X54-5/7.1, APC) and CD11b (M1/70, AF700) in PBS containing 1% BSA and 5 mM EDTA for 25 min at 4°C. For the adaptive immune cells, they were stained with CD11c (HL3, V450), Siglec-F (E50-2440, BV786), CD19 (6D5, AF488), CD45 (30-F11, PerCP-Cy5.5), Ly6G (1A8, PE-Cy7) and CD3 (17A2, AF700). Samples were analysed on a BD-Fortessa Flow Cytometer equipped with 50-mW 504-nm, 50-mW 488-nm, 50-mW 561-nm and 20-mW 633-nm lasers and an ND1.0 filter in front of the FSC photodiode. All antibodies were purchased from BD, Biolegend or eBioscience. Data were analyzed with FlowJo software (Tree Star).

**RNA isolation and quantitative RT-PCR**

Lung tissue was homogenized in TRIzol and RNA extraction performed according to manufacturer's instructions. After the chloroform step, the aqueous phase containing RNA was further processed using the RNeasy Mini Kit (QIAGEN) according to manufacturer's instructions. 2 µg RNA was reverse transcribed using a High-Capacity RNA-to-cDNA kit (Applied Biosystems) according to manufacturer's instructions. To quantify mRNA levels in lung tissue, quantitative RT-PCR reactions for *Oas1, Viperin* and *Ifnl* were performed using primers and probes as previously described (23). Analysis was performed using the QuantiTect Probe PCR Master Mix (QIAGEN) and the 7500 Fast real-Time PCR System (Applied Biosystems). For absolute quantification, the exact number of copies of the gene of interest was calculated using a plasmid DNA standard curve, and the results were normalized to levels of *Gapdh* (Applied Biosystems). For relative quantification, the expression of *Cxcl1, Cxcl10, hACE2, Mx1* and SARS-CoV-2 *N* and *E* gene was expressed relatively to the expression of *Gapdh*. First, the ΔCT (CT = cycle threshold) between the target gene and *Gapdh* was calculated for each sample, followed by calculation of $2^{-\Delta CT}$. Analysis was performed using 7500 Fast System SDS Software (Applied Biosystems).

**Chemokine and IFN detection**

For Peer Review

CCL2 and IFN-$\lambda$2/3 quantifications were performed on BAL fluid using mouse DuoSet ELISA (R&D Systems) according to the manufacturer's instructions. Data were acquired on a SpectraMax Plus plate reader (Molecular Devices) and analysed using SoftMax software (version 5.2). The concentration of IFN-α and IFN-β was measured in BAL fluid using the Mouse ProCartaPlex Immunoassay (Invitrogen) according to the manufacturer's instructions. Data were acquired and analysed with a Bio-Plex 200 system (Bio-Rad Laboratories).

**Statistical analysis**

Statistical analysis was performed using Prism 9.2 (Graph-Pad Software). One-way ANOVA with Tukey's post-hoc test was used to compare multiple groups. Data are expressed as mean ± SEM, and for all tests a value of $P < 0.05$ was considered significant. * $P < 0.05$, ** $P < 0.01$, *** $P < 0.005$, **** $P < 0.001$

### Acknowledgements

C. J. is supported by grants from UKRI-BBSRC (BB/V013831/1), Rosetrees Trust and Stoneygate Trust (M370 and M370-F1), Rosetrees Trust and The John Black Charitable Foundation (M956) and the Imperial College COVID-19 research fund. W.S.B., J.Z. and J.C.B are supported by the G2P-UK National Virology consortium funded by MRC/UKRI (grant ref: MR/W005611/1.). D.R.G., S.C.H. and Y.D are supported by a Wellcome Trust Portfolio grant (110579/Z/15/Z). For the purpose of open access, the authors have applied a CC BY public copyright license to any Author Accepted Manuscript version arising from this submission. The graphical abstract has been created using Biorender.com. We also thank the staff of St Mary's flow cytometry facility and the St Mary's animal facility for their assistance and members of the Johansson lab for scientific discussions.

### Author contribution

P.P.O. designed, performed, and analyzed the experiments and wrote the paper. M.G.M. and C.M. performed specific experiments and reviewed the paper. Y.D., D.R.G., S.C.H, O.H., and K.M.M. designed, manufactured and provided rAAV9-*hACE2* and rAAV9-*eGFP* and reviewed the paper, additionally Y.D. and K.M.M. performed specific experiments. J.Z., J.C.B. and W.S.B. provided advice and the SARS-CoV-2 WT isolate (D614G) and reviewed the paper. C.J. supervised the project, designed the experiments, and wrote the paper.

### Conflict of interest

For Peer Review

The authors declare no commercial or financial conflict of interest.

### Data availability statement

The data that support the findings of this study are available from the corresponding author upon reasonable request.

### Ethics approval

All animal experiments were reviewed and approved by the Animal Welfare and Ethical Review Board (AWERB) at Imperial College London and approved by the UK Home Office in accordance with the Animals (Scientific Procedures) Act 1986 Amendment Regulations (PPL P3AFFF0DD Johansson).

For Peer Review

**Figure 1: Increased viral load, weight loss and lower expression of ISGs in *Ifnar1*$^{-/-}$ mice during infection with SARS-CoV-2**

A) Recombinant Adeno-associated virus (rAAV) containing human angiotensin converting enzyme 2 (h*ACE2*) or *eGFP* genes was administered intranasally to *Ifnar1*$^{-/-}$ or wildtype (WT) mice (1x10$^{11}$ Dnase resistant gene copies/mouse). 20 days later mice were intranasally infected with SARS-CoV-2 (D614G, 2x10$^{6}$ PFU/mouse). Lungs and bronchoalveolar lavage (BAL) were harvested at 2, 4 and 8 days post infection (d.p.i). B) Expression of *hACE2* in lung tissue relative to *Gapdh*, measured by RT-PCR before infection (d20 post transduction with rAAV). WT/rAAV-e*GFP* n = 3, *Ifnar1*$^{-/-}$ /rAAV-e*GFP* n = 4, WT/rAAV-*hACE2* n = 4, *Ifnar1*$^{-/-}$ /rAAV-*hACE2* n = 5). C) Weight loss post infection with SARS-CoV-2. D) Viral load measured by plaque assay on Vero cells overexpressing hACE2 and TMPRESS2. E) Expression of SARS-CoV-2 *N gene* (nucleocapsid phosphoprotein) and *E gene* (envelope protein) in lung tissue relative to *Gapdh*, measured by RT-PCR. F) Gene expression analysis of IFN stimulated genes (ISG) *Cxcl10*, *Mx1*, *Oas1* and *Viperin* measured by RT-PCR, relative to expression of *Gapdh* or total copy numbers normalized to expression of *Gapdh (Oas1* and *Viperin).* Data are shown as mean ± SEM. B) WT/rAAV-e*GFP* n = 3, WT/rAAV-*hACE2* n = 4, *Ifnar1*$^{-/-}$ /rAAV-*hACE2* n = 5 C – F) Two independent experiments per time point, data pooled, n = 6-8 per group. One Way ANOVA + Tukey's multiple comparison test per time point; * indicates significant difference between WT rAAV-*hACE2* and *Ifnar1$^{-/-}$* rAAV-*hACE2*, * P < 0.05, ** P < 0.01, *** p < 0.005, **** p < 0.001.

**Figure 2: Increased neutrophil recruitment to airways in IFNAR1-deficient mice during SARS-COV-2 infection**

A) Gene expression of *Cxcl1* in lung tissue relative to *Gapdh* at 2, 4 and 8 d.p.i. with SARS-CoV-2 (D614G), measured by RT-PCR. B) Proportions of live, CD45$^{+}$ and total numbers of neutrophils in BAL at 2 d.p.i. C) Representative flow cytometry plots of lung cells gated on live, CD45$^{+}$ Ly6G$^{+}$ D) Proportions of live, CD45$^{+}$ and total numbers of neutrophils in lung tissue at 2, 4 and 8 d.p.i. Data are shown as mean ± SEM. Two independent experiments per time point, data pooled, n = 6-8 per group. One Way ANOVA + Tukey's multiple comparison test per time point; * indicates significant difference between *hACE2*-WT and *hACE2-Ifnar1$^{-/-}$*, * P < 0.05, ** P < 0.01, *** P < 0.005, **** P < 0.001.

**Figure 3: Type I interferon signaling deficiency results in dysregulated inflammatory myeloid cell recruitment during SARS-CoV-2 infection**

A) Protein expression of CCL2 in BAL fluid at 2, 4 and 8 d.p.i. measured by ELISA. B) Proportions of live, CD45$^{+}$ and total numbers of CD64$^{+}$ CD11b$^{+}$ inflammatory myeloid cells in lung tissue at 2, 4 and 8 d.p.i. C) Representative flow cytometry plots of lung cells gated on live, CD45$^{+}$ Ly6G$^{-}$ SigF $^{-}$ CD11b$^{+}$. D) Proportions of live, CD45$^{+}$ and total numbers of CD64$^{+}$ CD11b$^{+}$ Ly6C$^{+}$ inflammatory myeloid cells in lung tissue at 2, 4 and 8 d.p.i. E) Proportions of live, CD45$^{+}$ and total numbers of CD64$^{+}$ CD11b$^{+}$ Ly6C$^{-}$ inflammatory myeloid cells in lung tissue at 2, 4, and 8 d.p.i. Data are shown as mean ± SEM. Two independent experiments per time point, data pooled, n = 6-8 per group. One Way ANOVA + Tukey's multiple comparison test per time point; * indicates significant difference between *hACE2*-WT and *hACE2-Ifnar1$^{-/-}$*, * P < 0.05, ** P < 0.01, *** P < 0.005, **** P < 0.001. Dotted line = limit of detection.

For Peer Review

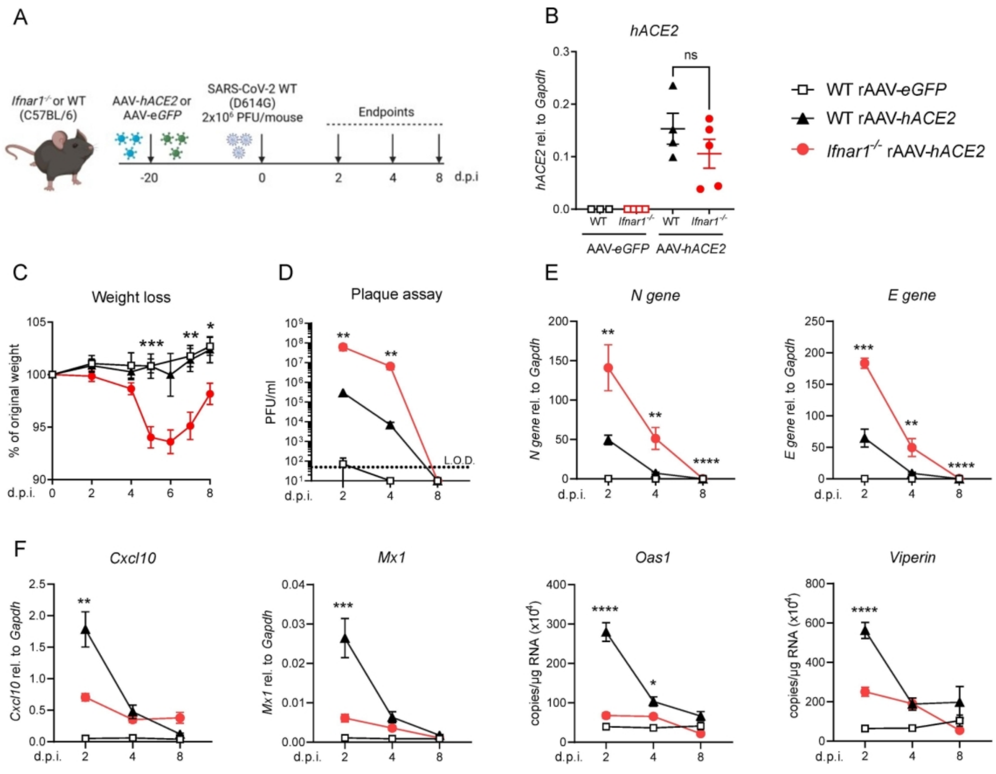

Fig1 eps format

161x122mm (300 x 300 DPI)

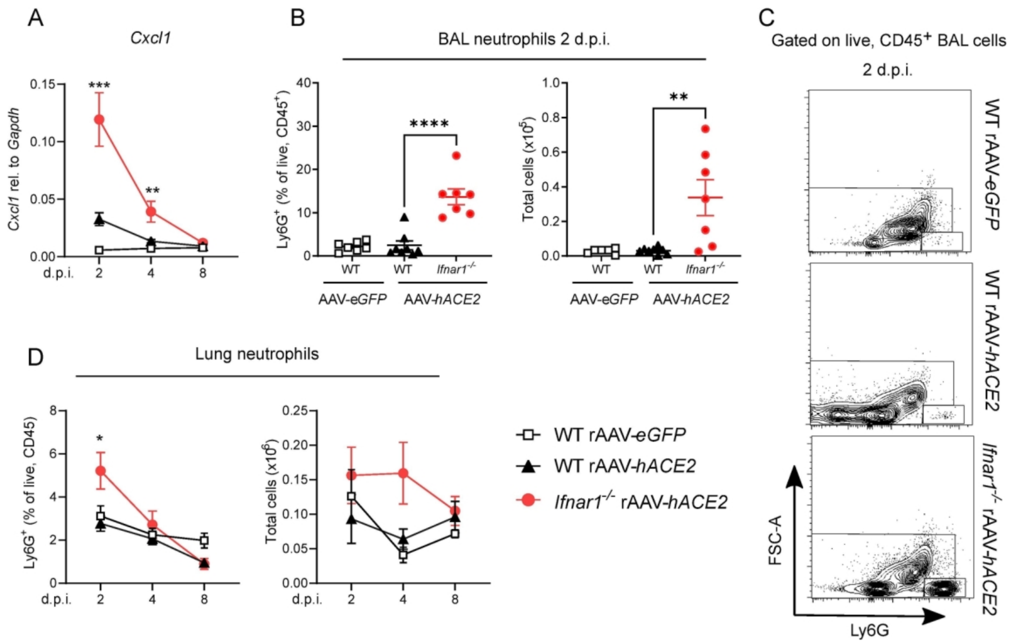

Fig2 eps format

147x93mm (300 x 300 DPI)

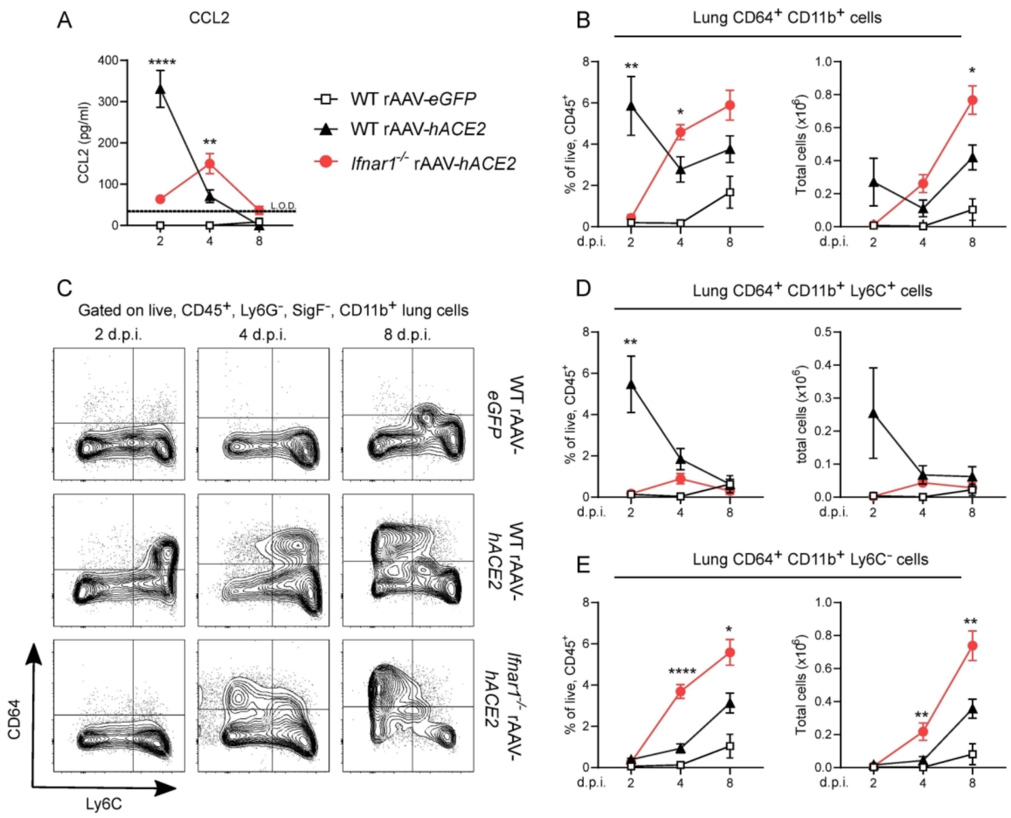

Fig3 eps format

154x124mm (300 x 300 DPI)

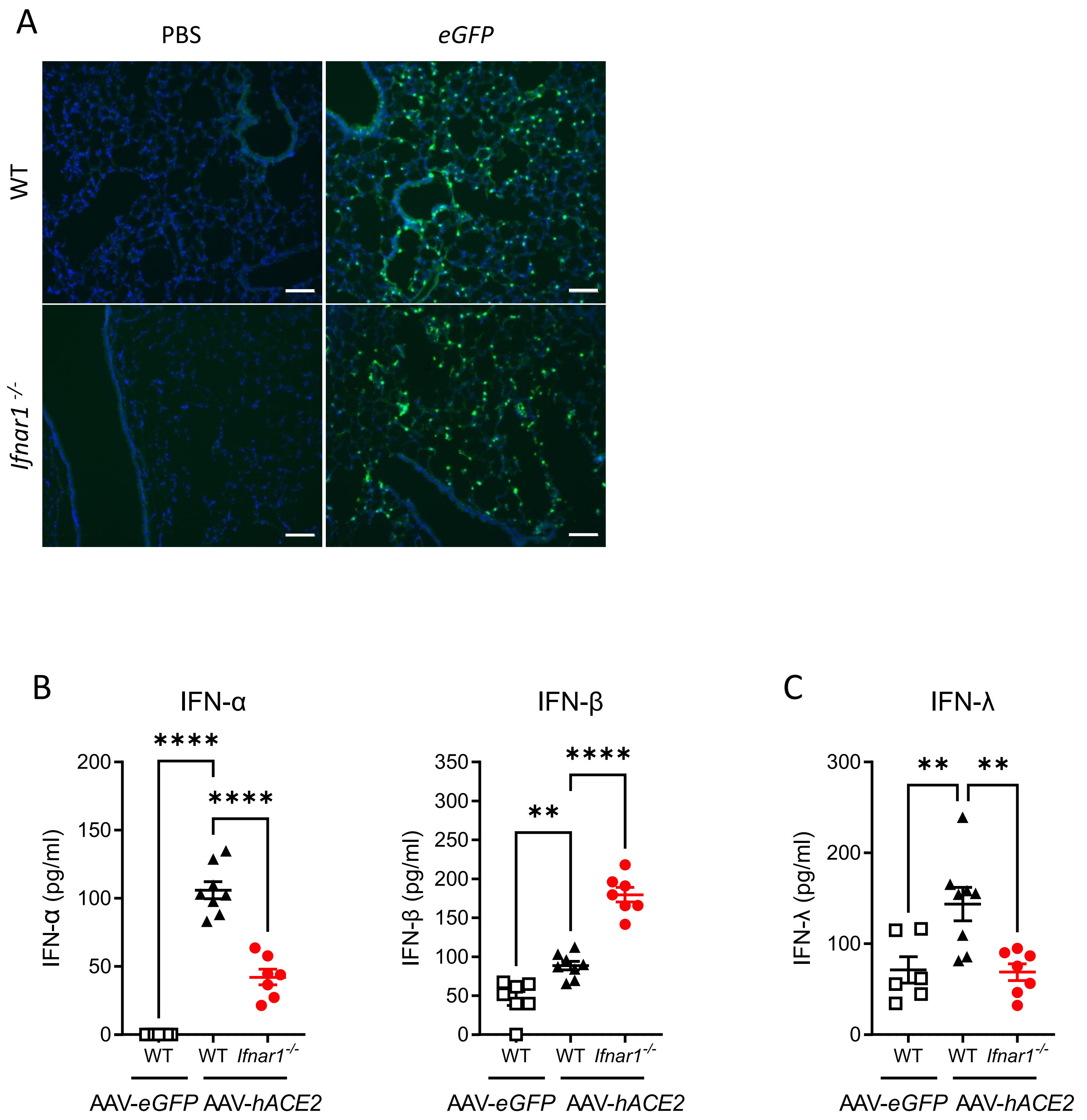

**Supplemental Figure 1: Validation of *hACE2* transduction and interferon expression upon SARS-CoV-2 infection**

A) *eGFP* signal in lung cryosections of WT or *Ifnar1*$^{-/-}$ transduced with rAAV-*eGFP* (20 days post transduction), or vehicle (PBS). Representative images shown for each group (2-3 mice per group). Scale bar = 100 µm. B) Expression of IFN-α and IFN-β in BAL fluid at 2 d.p.i. measured by Luminex Procarta assay. C) Expression of IFN-λ in BAL fluid at 2 d.p.i. measured by ELISA. Data are shown as mean ± SEM. Two independent experiments per time point, data pooled, n = 6-8 per group. One Way ANOVA + Tukey's multiple comparison test; * indicates significant difference between WT rAAV-*hACE2* and *Ifnar1*$^{-/-}$ rAAV-*hACE2*, * $P < 0.05$, ** $P < 0.01$, *** $P < 0.005$, **** $P < 0.001$.

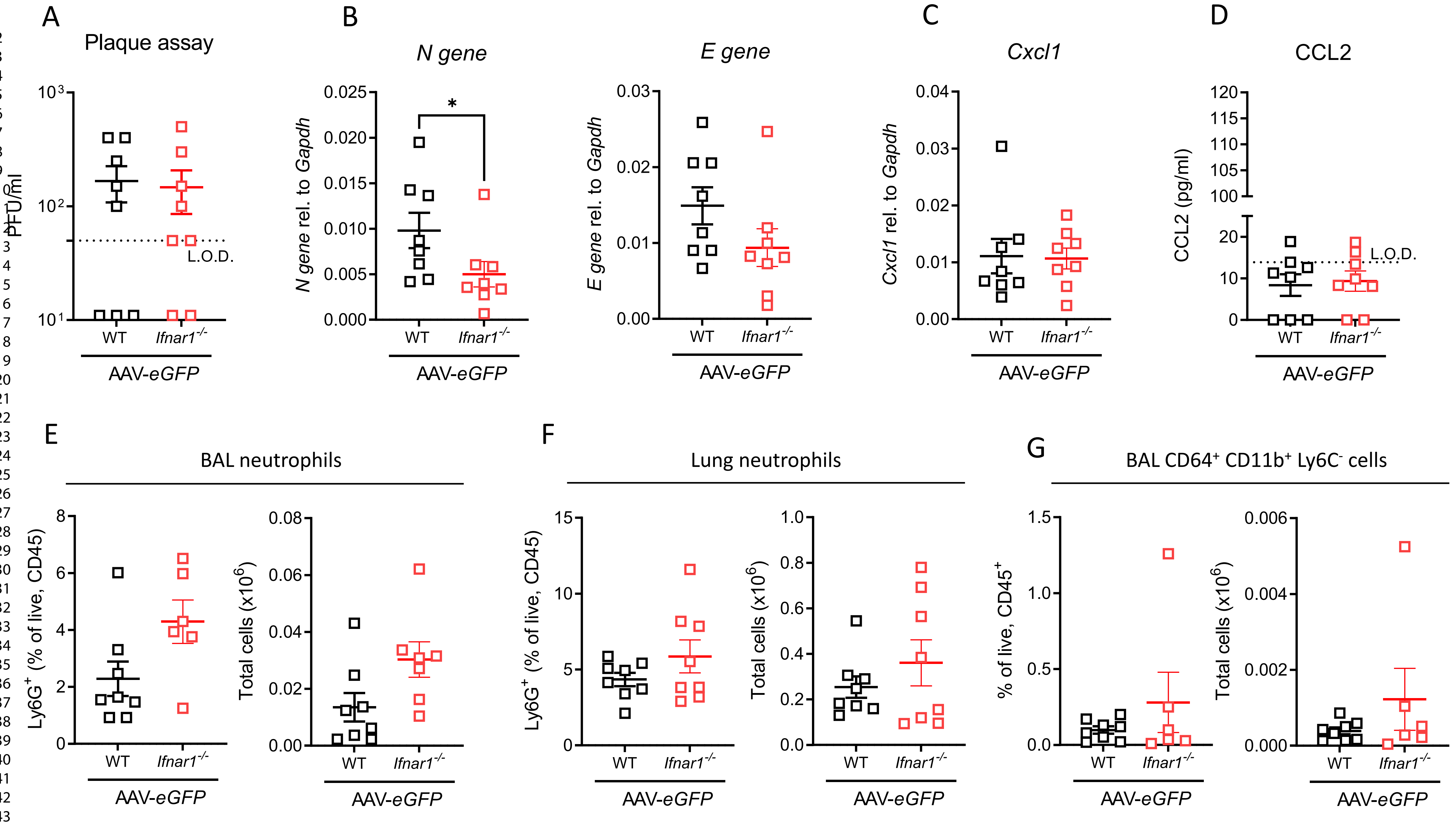

**Supplemental Figure 2: Baseline viral load and immune response at 0.75 d.p.i. with SARS-CoV-2 in rAAV-*eGFP* transduced WT and *Ifnar1*$^{-/-}$ mice**

WT and *Ifnar1*$^{-/-}$ mice were transduced with rAAV-*eGFP* and after 20 days infected with with SARS-CoV-2 (D614G, 2x10$^6$ PFU/mouse). BAL and lungs were analysed at 0.75 d.p.i. A) Viral load measured by plaque assay on Vero cells overexpressing *hACE2* and *TMPRESS2* B) Expression of SARS-CoV-2 *N gene* (nucleocapsid phosphoprotein) and *E gene* (envelope protein) in lung tissue relative to *Gapdh*, measured by RT-PCR C) Gene expression of *Cxcl1* in lung tissue relative to *Gapdh. D*) Protein expression of CCL2 in BAL fluid measured by ELISA. E-F) Proportions of live, CD45$^+$ and total numbers of neutrophils in BAL (E) and lung (F) at 0.75 d.p.i. G) Proportions of live, CD45$^+$ and total numbers of CD64$^+$ CD11b$^+$ Ly6C$^-$ monocytes in BAL at 0.75 d.p.i. Data are shown as mean ± SEM. Two independent experiments, data pooled, n = 6-8 per group. One Way ANOVA + Tukey's multiple comparison test; * indicates significant difference between WT AAV-*hACE2* and *Ifnar1*$^{-/-}$ AAV-*hACE2*, * P < 0.05.

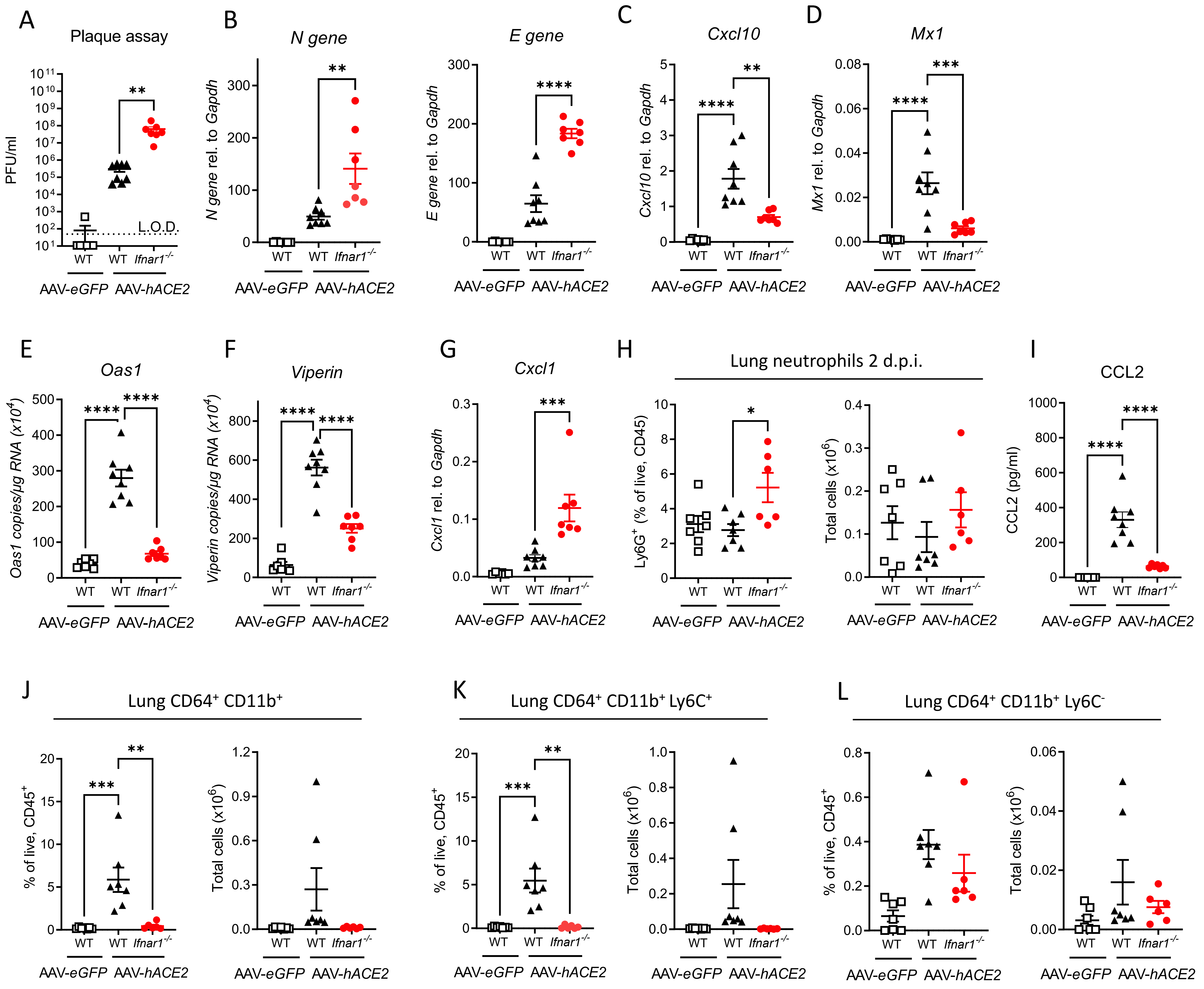

**Supplemental Figure 3: Viral load and immune responses at 2 d.p.i. with SARS-CoV-2 in rAAV-*hACE2* mouse model**
WT and *Ifnar1*$^{-/-}$ mice were transduced with rAAV-*eGFP* or rAAV-*hACE2* and after 20 days infected with with SARS-CoV-2 (D614G, 2x10$^{6}$ PFU/mouse). BAL and lungs were analysed at 2 d.p.i A) Viral load measured by plaque assay on Vero cells overexpressing hACE2 and TMPRESS2 B) Expression of SARS-CoV-2 *N gene* (nucleocapsid phosphoprotein) and *E gene* (envelope protein) in lung tissue relative to *Gapdh*, measured by RT-PCR C-F) Gene expression analysis of IFN stimulated genes (ISG) *Cxcl10 (C)*, *Mx1 (D)*, *Oas1 (E)* and *Viperin (F)* measured by RT-PCR, relative to expression of *Gapdh* or total copy numbers normalized to expression of *Gapdh (Oas1* and *Viperin). G)* Gene expression of *Cxcl1* in lung tissue relative to *Gapdh*. H) Protein expression of CCL2 in BAL fluid measured by ELISA. I) Proportions of live, CD45$^{+}$ and total numbers of neutrophils in lung tissue J - L) Proportions of live, CD45$^{+}$ and total numbers of CD64$^{+}$ CD11b$^{+}$ inflammatory myeloid cells (J), CD64$^{+}$CD11b$^{+}$Ly6C$^{+}$ cells (K) and CD64$^{+}$CD11b$^{+}$Ly6C$^{-}$ cells (L) in lung tissue. Data are shown as mean ± SEM. Tissues were collected at 2 d.p.i. Two independent experiments per time point, data pooled, n = 6-8 per group. One Way ANOVA + Tukey's multiple comparison test; * indicates significant difference between WT AAV-*hACE2* and *Ifnar1*$^{-/-}$ AAV-*hACE2*, * $P < 0.05$, ** $P < 0.01$ , *** $P < 0.005$, **** $P < 0.001$.

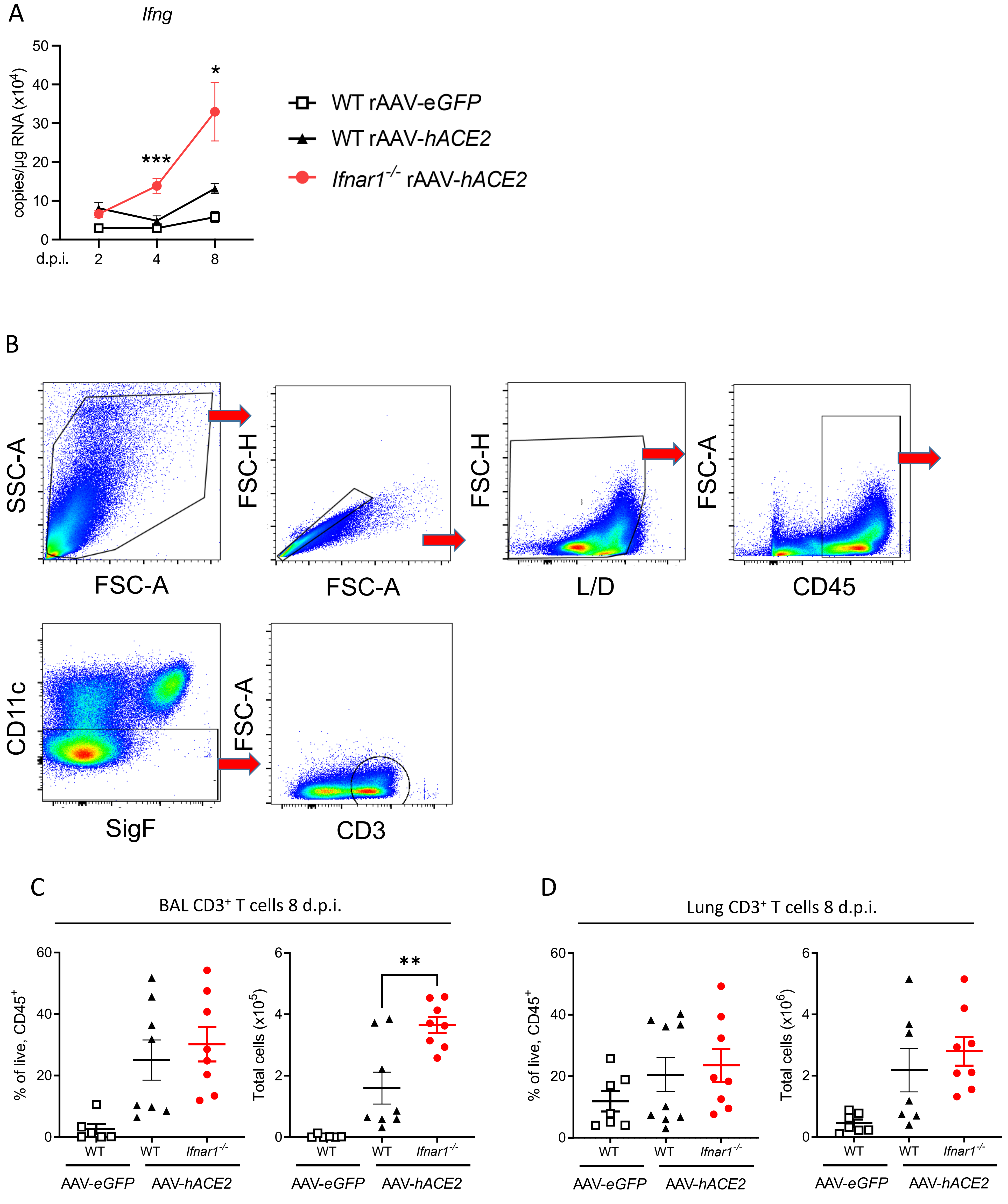

**Supplemental Figure 4: Interferon-ɣ and T cell recruitment to the lungs upon infection SARS-CoV-2 in rAAV-*hACE2* mouse model**

A) Gene expression analysis of *Ifng* measured in lung homogenates by RT-PCR, total copy numbers normalized to expression of *Gapdh* at 2, 4 and 8 days. B) Gating strategy to detect $CD3^+$ T cells in BAL and lung 8 days after infection with SARS-CoV-2. C – D) Proportions of live, $CD45^+$ and total numbers of T cells in BAL (C) and lung (D) at 8 d.p.i. Data are shown as mean ± SEM. Two independent experiments per time point, data pooled, n = 6-8 per group. One Way ANOVA + Tukey's multiple comparison test per time point; * $P < 0.05$, ** $P < 0.01$ , *** $P < 0.005$, **** $P < 0.001$.

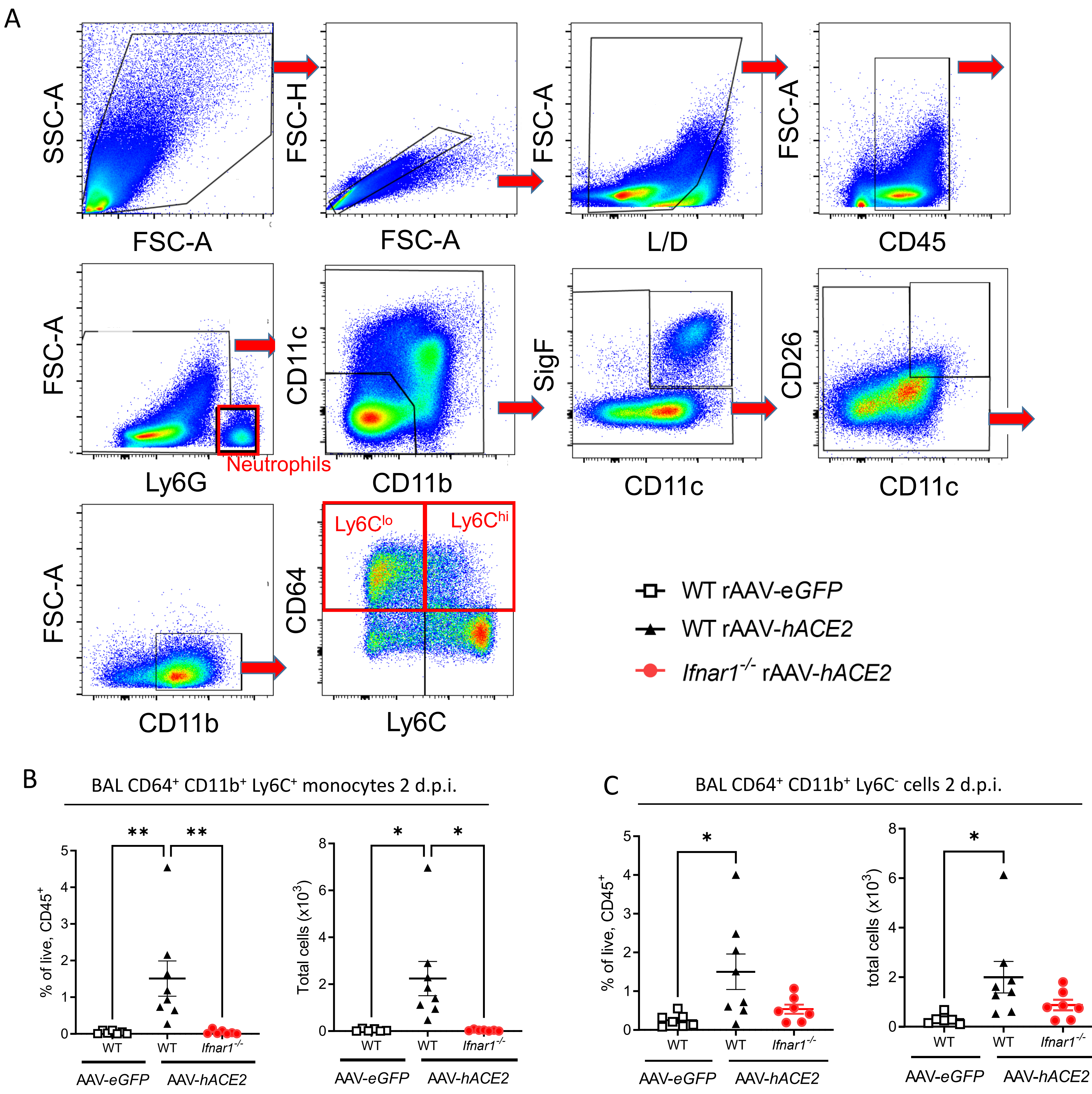

**Supplemental Figure 5: Myeloid cell recruitment into the airways upon infection SARS-CoV-2 in rAAV-*hACE2* mouse model**

A) Gating strategy to detect innate immune cell populations in BAL and lung after infection with SARS-CoV-2. Populations of interest (highlighted in red) are Ly6G$^+$ neutrophils, CD11b$^+$CD64$^+$Ly6C$^-$ and CD11b$^+$CD64$^+$Ly6C$^+$ inflammatory myeloid cells. B-C) Proportions of live, CD45$^+$ and total numbers of CD64$^+$ CD11b$^+$ Ly6C$^+$ monocytes (C) and CD64$^+$ CD11b$^+$ Ly6C$^-$ monocytes (D) in BAL at 2 d.p.i. Data are shown as mean ± SEM. Two independent experiments per time point, data pooled, n = 6-8 per group. One Way ANOVA + Tukey's multiple comparison test; * P < 0.05, ** P < 0.01 , *** P < 0.005, **** P < 0.001.

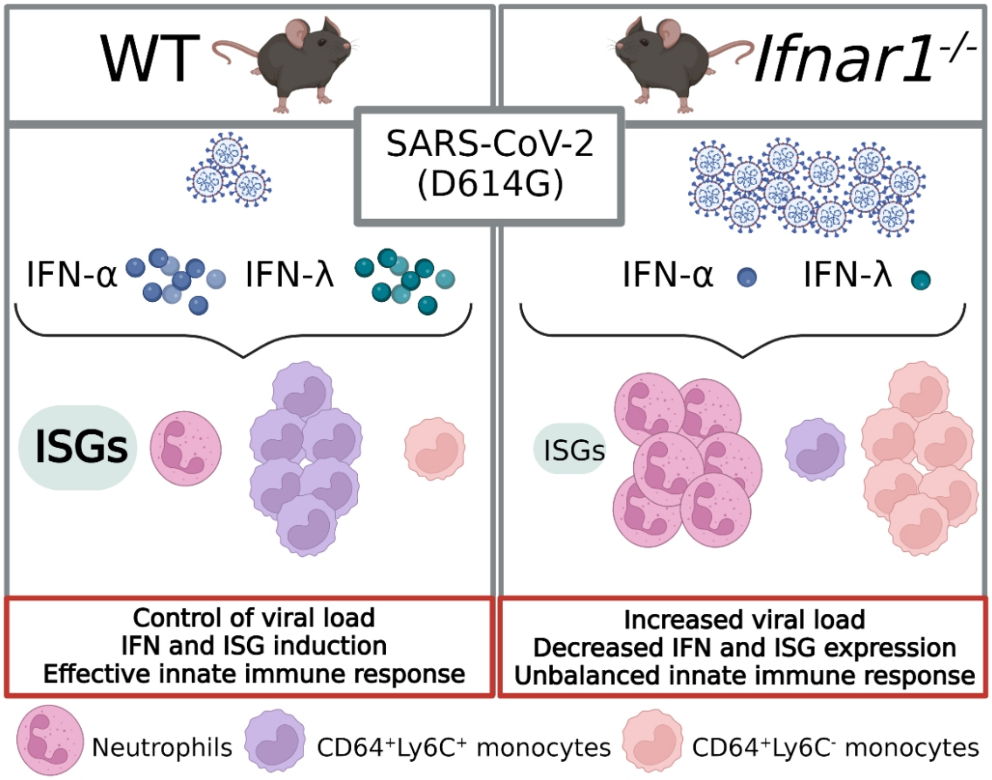

127x101mm (236 x 236 DPI)

During SARS-CoV-2 infection in mice, IFNAR1-deficiency results in dysregulated innate immune responses, such as increased neutrophil recruitment to the lung and an altered monocyte population profile. Together with decreased expression of anti-viral effector interferon-stimulated genes *Mx1*, *Oas1* and *Viperin* this results in higher viral loads and overall worsened disease phenotype.

For Peer Review

## Type I interferon signaling deficiency results in dysregulated innate immune responses to SARS-CoV-2 in mice

**Authors:** Patricia P. Ogger[1], Minerva Garcia Martín[1], Christina Michalaki[1], Jie Zhou[2], Jonathan C. Brown[2], Yue Du[3], Kamran M. Miah[3], Omar Habib[3], Stephen C. Hyde[3], Deborah R. Gill[3], Wendy S. Barclay[2], Cecilia Johansson[1]

[1]Section of Respiratory Infections, National Heart and Lung Institute, Imperial College London, St Mary's Campus, W2 1PG

[2]Department of Infectious Disease, Imperial College London, St Mary's Campus, W2 1PG

[3]Radcliffe Department of Medicine (NDCLS), University of Oxford, OX3 9DU

Lead contact: Dr Cecilia Johansson, c.johansson@imperial.ac.uk

St Mary's Campus, National Heart and Lung Institute, Imperial College London, W2 1PG, UK.

For Peer Review

### Abstract:

SARS-CoV-2 is a newly emerged coronavirus, causing the global pandemic of respiratory coronavirus disease (COVID-19). The type I interferon (IFN) pathway is of particular importance for anti-viral defence and recent studies identified that type I IFNs drive early inflammatory responses to SARS-CoV-2. Here, we use a mouse model of SARS-CoV-2 infection, facilitating viral entry by intranasal recombinant Adeno-Associated Virus (rAAV) transduction of *hACE2* in wildtype (WT) and type I IFN-signalling-deficient (*Ifnar1*$^{-/-}$) mice, to study type I IFN signalling deficiency and innate immune responses during SARS-CoV-2 infection. Our data show that type I IFN signaling is essential for inducing anti-viral effector responses to SARS-CoV-2, control of virus replication and to prevent enhanced disease. Furthermore, *hACE2-Ifnar1*$^{-/-}$ mice had increased gene expression of the chemokine *Cxcl1* and airway infiltration of neutrophils as well as a reduced and delayed production of monocyte-recruiting chemokine CCL2. *hACE2-Ifnar1*$^{-/-}$ mice showed altered recruitment of inflammatory myeloid cells to the lung upon SARS-CoV-2 infection, with a shift from Ly6C$^{+}$ to Ly6C$^{-}$ expressing cells. Together, our findings suggest that type I IFN deficiency results in a dysregulated innate immune response to SARS-CoV-2 infection.

### Keywords:

Innate Immune Response/ type I IFN / In vivo / SARS-CoV-2/ myeloid cells

Wiley - VCH

For Peer Review

### Introduction

The ongoing coronavirus disease (COVID-19) pandemic caused by severe acute respiratory syndrome-coronavirus 2 (SARS-CoV-2) has resulted in over 400 million cases in the first two years of the pandemic. The estimated fatality rate lies between 1-2%, however this is considerably higher for elderly patients over 80 years of age (~10%) and nursing home residents (>20%) (1). Type I interferons (IFNs) are one of the first responses elicited against viral infection and they induce anti-viral defense mechanisms by binding to the IFN-α/β receptor (IFNAR) and signaling through the JAK-STAT pathway. This induces expression of IFN stimulated genes (ISG), resulting in expression of anti-viral effector proteins that restrict viral replication and activation of immune cells via induction of chemokine and cytokine production (2), including CXCL10 and CCL2 (3). Early *in vitro* studies using human bronchial epithelial cell lines infected with SARS-CoV-2 showed decreased production of type I and III IFNs coupled with low anti-viral defense signals and a pro-inflammatory environment compared to infection with influenza A virus (IAV) (4). Furthermore, in severe and critically ill COVID-19 patients, an impaired type I IFN response has been observed, resulting in decreased viral clearance (5). A lack of an efficient type I IFN response in these patients is in part due to inborn errors of type I IFN immunity (6) or circulating auto-antibodies neutralizing type I IFNs (7). Also, a recent animal study has identified that type I IFN signaling is required for the recruitment of pro-inflammatory cells into the lungs following SARS-CoV-2 infection (8). These findings highlight the importance of functional type I IFN responses for anti-viral defenses against SARS-CoV-2.

Using a mouse model of SARS-CoV-2 infection, facilitated by intranasal recombinant Adeno-Associated Virus (rAAV) induced expression of human angiotensin converting enzyme 2 (hACE2), this study investigated the dynamics of innate immune responses to infection with SARS-CoV-2 in the context of type I IFN signaling impairment. Overall, the data show that type I IFN signaling is essential to induce anti-viral responses and control viral replication and disease severity during SARS-CoV-2 infection. Furthermore, type I IFN signaling-deficient mice show dysregulated innate immune responses to SARS-CoV-2 infection, marked by increased neutrophil recruitment into the airways and delayed recruitment of myeloid inflammatory cells.

### Results and discussion

To investigate the dynamics of innate, anti-viral immune responses to SARS-CoV-2 infection in the context of type I IFN-signaling deficiency, 8–12-week-old C57BL/6 wildtype (WT) or *interferon alpha receptor-1*$^{-/-}$ (*Ifnar1*$^{-/-}$) mice were intranasally transduced with rAAV9 containing either *hACE2* or *eGFP* (control) as published recently (9), followed by intranasal infection with $2x10^6$ PFU SARS-CoV-2

For Peer Review

(D614G, first wave isolate) 20 days later and study endpoints were at 2, 4 and 8 days post infection (d.p.i.) (Figure 1A). Gene expression analysis of *hACE2* in lung tissue 20 days post administration of rAAV9 (before infection) showed similar expression in WT and *Ifnar1*$^{-/-}$ mice relative to *Gapdh* (Figure 1B). Cryo-sectioning of lung tissue 20 days post administration of rAAV9-*eGFP* (before infection) furthermore showed similar distribution of *eGFP* in WT and *Ifnar1*$^{-/-}$ mice (Supp. Figure 1A), suggesting similar rAAV transduction efficacy in both groups of mice. Upon infection with SARS-CoV-2, *hACE2-Ifnar1*$^{-/-}$ mice showed increased weight loss, peaking at 6 d.p.i., compared with *hACE2*-WT mice (Figure 1C). Furthermore, plaque assays on Vero cells overexpressing SARS-CoV-2 binding receptors ACE2 and transmembrane protease serine 2 precursor TMPRSS2 (VAT cells) showed that IFNAR1-deficiency results in significantly higher viral loads at day 2 and 4 post infection with SARS-CoV-2 compared to *hACE2*-WT mice (Figure 1D). Gene expression of SARS-CoV-2 nucleocapsid (N) and envelope (E) genes was significantly higher in *hACE2-Ifnar1*$^{-/-}$ mice compared with *hACE2*-WT mice from 2 d.p.i. (Figure 1E and Supp. Figure 2B). Since AAV-e*GFP* transduced *Ifnar1*$^{-/-}$ mice did not become infected with SARS-CoV-2 and did overall not differ significantly from AAV-e*GFP* transduced WT mice (Supp. Figure 2), this group was not included in all experiments to reduce animal numbers. Together, these data show increased viral load in *hACE2* expressing IFNAR1-deficient mice upon SARS-CoV-2 infection, measured both by plaque assay and N and E gene expression. Previous studies have shown similar trends (8), although differences between WT and *Ifnar1*$^{-/-}$ are more pronounced in the model used here. This could be due to several factors: here, Vero cells overexpressing hACE2 and TMPRSS2 were used for plaque assays, allowing for better viral replication, while the isolate used for infection was hCoV-19/England/IC19/2020, which harbors the D614G spike mutation as opposed to USA-WA1/2020, enhancing viral replication (10). Together, these factors might explain why we detected higher viral loads and more prominent differences emerged between *hACE2*-WT and *hACE2-Ifnar1*$^{-/-}$ mice.

For Peer Review

To investigate the impact of impaired type I IFN signaling during SARS-CoV-2 infection, we first assessed type I IFN expression upon infection. At 2 d.p.i. both IFN-α and IFN-β were significantly increased in BAL fluid of *hACE2*-WT compared to e*GFP*-WT mice (Supp. Figure 1B). In the *hACE2-Ifnar1*$^{-/-}$ group, expression of IFN-α was significantly lower, while IFN-β levels were higher compared to the *hACE2*-WT group. We next investigated ISG expression after SARS-CoV-2 infection, since in *Ifnar1*$^{-/-}$ mice limited amounts of type I IFN cytokines can be produced but cannot signal for downstream ISGs induction. Chemokine *Cxcl10* and anti-viral effectors *Mx1, Oas1* and *Viperin* were quantified. The expression of these ISGs was increased in *hACE2*-WT mice upon infection with SARS-CoV-2 at 2 d.p.i., however in *hACE2-Ifnar1*$^{-/-}$ mice, ISG expression was significantly reduced, but not completely absent (Figure 1F and Supp. Figure 3 C-F). These results suggest an initial increase of IFN-β in *hACE2-Ifnar1*$^{-/-}$ mice, which may be due to higher viral titers, but that is not translated into ISG expression due to the lack of signaling through the IFNAR1. We therefore investigated the expression of type III IFNs, IFN-λ2-3, which can contribute to ISG expression. IFN-λ expression was induced upon infection in *hACE2*-WT mice at 2 d.p.i. while remained at baseline levels in the *hACE2-Ifnar1*$^{-/-}$ mice (Supp. Figure 1C). Furthermore, *Ifng* gene expression was significantly increased in *hACE2- Ifnar1*$^{-/-}$ mice later during the infection, by 4 d.p.i. (Supp. Figure 4A). This correlated with CD3$^{+}$ T cell recruitment to the airways (Supp. Figure 4B-D). Our data suggest that limited levels of type I or III IFNs are produced early during infection in the *Ifnar1*$^{-/-}$ mice resulting in some ISG expression but overall, these data suggest that type I IFN signaling is the main driver for inducing cell intrinsic anti-viral responses.

We next assessed the gene expression of inflammatory mediators and found that expression of the chemokine *Cxcl1*, which is not dependent on type I IFN signaling (3), was increased in *hACE2-Ifnar1*$^{-/-}$ mice at 2 and 4 d.p.i. compared to *hACE2*-WT (Figure 2A and Supp. Figure 3G). As CXCL1 plays an essential role in early host immune responses by recruiting neutrophils (11), we next analysed infiltration of neutrophils (gated as live, CD45$^{+}$, Ly6G$^{+}$, Supp. Figure 5A) into the airways at 2 d.p.i. In line with highly increased gene expression of *Cxcl1*, neutrophil recruitment to the airways (BAL) was significantly increased in *hACE2-Ifnar1*$^{-/-}$ mice at 2 d.p.i., both proportional of leukocytes (CD45$^{+}$ cells) and in total numbers (Figure 2B and C). This was recapitulated in lung tissue with increased proportions of neutrophils in type I IFN signaling-impaired mice at 2 d.p.i. (Figure 2D and Supp. Figure 3H), decreasing over time. Taken together, these findings suggest that during SARS-CoV-2 infection, type I IFN signaling deficiency results in increased neutrophil recruitment via CXCL1, thereby contributing to a pro-inflammatory environment. Indeed, *Cxcl1* is also increased in *Ifnar1*$^{-/-}$ mice during influenza A with secondary pneumococcal infection (12), but decreased during RSV infection in mice (13), highlighting a pathogen specific CXCL1 response. Furthermore, since we show similar trends for viral load and neutrophil recruitment upon SARS-CoV-2 infection (both significantly increased in *hACE2-Ifnar1*$^{-/-}$ mice), which is a mechanism present in other respiratory viral infections such as respiratory syncytial virus (RSV) (13, 14), it will be important to further investigate the link between neutrophil recruitment and viral load in this model.

Since monocyte recruitment to the airways and lungs is key to early host responses to viral infection, we next investigated the expression of monocyte recruiting chemokine CCL2 and the recruitment of inflammatory myeloid cells. CCL2 protein expression was increased in BAL fluid of *hACE2*-WT mice at 2 d.p.i. with SARS-CoV-2 (Supp. Figure 3I). However, in *hACE2-Ifnar1*$^{-/-}$ mice CCL2 expression was significantly lower at 2 d.p.i., peaking at 4 d.p.i. at lower levels than in IFNAR1-sufficient mice (Figure 3A). These findings are in line with a report identifying early CCR2 signaling essential to restrict viral

For Peer Review burden in the lung in a mouse model of SARS-CoV-2 infection (15). The recruitment of $CD64^+CD11b^+$ inflammatory myeloid cells to the lung followed similar kinetics, as in *hACE2*-WT mice proportions were highest at 2 d.p.i. and subsequently decreased, while in IFNAR1-deficient mice proportions and total numbers of $CD64^+CD11b^+$ inflammatory myeloid cells strongly increased between 2 and 4 d.p.i. and were highest at 8 d.p.i. (Figure 3B and Supp. Figure 3J). We next assessed expression of the monocyte/macrophage differentiation antigen Ly6C within this population, since previous studies reported the infiltration of $CD64^+CD11b^+Ly6C^+$ inflammatory myeloid cells into the lung during SARS-CoV-2 infection (8, 15). This showed highly increased proportions of $CD64^+CD11b^+Ly6C^+$ in *hACE2*-WT but not IFNAR1-deficient mice at 2 d.p.i. in the BAL (Supp. Figure 5D) and lung (Figure 3C-D and Supp. Figure 3K), suggesting type I IFN dependency for recruitment. However, as we have previously shown that Ly6C is gradually downregulated on monocytes during response to respiratory viral infection (16), we also analyzed $CD64^+CD11b^+$ $Ly6C^-$ cells. The presence of $CD64^+CD11b^+$ $Ly6C^-$ inflammatory myeloid cells in the airways was not type I IFN signaling dependent, since both proportions and total numbers were significantly increased in *hACE2-Ifnar1*$^{-/-}$ mice at 4 and 8 d.p.i. (Figure 3C and E), while at 2 d.p.i. in the airways no significant differences emerged (Supp. Figure 5F). This accounts for the delayed emergence of inflammatory myeloid cells in the lung during type I IFN signaling impairment shown in Figure 3B and overall indicates altered recruitment dynamics of inflammatory myeloid cells. Taking these data together, our model recapitulates the deficiency of type I interferon responses seen in severe SARS-CoV-2 infection, which in patients is marked by decreased IFN-α, type I IFN activity and ISG score, as well as neutrophilia and increased CCL2 (5). Our data suggest that the lack of type I IFN signaling results in dysregulated innate immune responses in the lung during SAR-CoV-2 infection.

### Concluding Remarks

In summary, using a mouse model of SARS-CoV-2 infection we show that type I IFN signaling is essential for inducing anti-viral effector responses, control of virus replication and disease severity. Our data indicate that type I IFN signaling-deficient mice express increased levels of *Cxcl1* in the lung and increased infiltration of neutrophils to the airways compared to WT controls. Furthermore, we found reduced and delayed production of CCL2 and altered recruitment of inflammatory myeloid cells during IFNAR1-deficiency. This, together with an increased viral burden is associated with more severe disease in type I IFN signaling-deficient mice. The data shown here will be valuable for better understanding how impaired type I IFN signaling drives SARS-CoV-2 pathology and disease severity, which is highly relevant considering the large contribution of impaired type I IFN responses on life-threatening SARS-CoV-2 infections (6, 7) and deaths (17) and for the development of type I IFN-based treatment options for COVID-19 in vulnerable populations. To conclude, our findings show that type I

For Peer Review

IFN deficiency results in dysregulated innate immune responses to SARS-CoV-2 infection in the rAAV-*hACE2* mouse model.

### Materials and Methods

#### Mice

C57BL/6 mice were purchased from Charles River UK Inc. *Ifnar1*$^{-/-}$ mice on a C57BL/6 background were bred in-house. All mice were bred and maintained in pathogen-free conditions and 8–12-week-old mice were used for experiments. All animal experiments were reviewed and approved by the Animal Welfare and Ethical Review Board (AWERB) at Imperial College London and approved by the UK Home Office in accordance with the Animals Act 1986 (Scientific Procedures) and ARRIVE guidelines. Both male and female mice were used for experiments after excluding sex bias in preliminary experiments. All experiments were performed twice, independently, per time point.

For Peer Review

#### rAAV vector production

The production, purification, and titration of rAAV2/9-*eGFP* or *hACE2* vectors were performed as previously described (9). Briefly, the respective rAAV vector was produced by polyethylenimine (PEI, PolySciences)-based triple transfection of human embryonic kidney (HEK) 293T/17 cells (ATCC, CRL-11268). The AAV plasmids transfected included the Adenovirus helper plasmid (pAdDeltaF6), AAV Rep-Cap pAAV2/9 plasmid and the transgene plasmid. The transgene plasmid containing *eGFP* or *hACE2* was engineered to include a lung-optimized hCEFI (human Cytomegalovirus enhancer/elongation factor 1 alpha) promoter (18), Woodchuck Hepatitis Virus Post-transcriptional Regulatory Element (WPRE) (19) and mir142-3pT (20). rAAV particles were concentrated and formulated into PBS using 100 kDa Ultra centrifugal filters (Amicon, Merck) after iodixanol gradient centrifugation. Physical titre (DNase-resistant genome copies, DRGC/mL) was determined by quantitative polymerase chain reaction (qPCR) analysis with primers and a probe against WPRE (21). Purity of vectors was confirmed by analyzing 20 µl of diluted vector on 4-12% SDS polyacrylamide gels, where total protein was visualized using Coomassie stain according to the manufacturer's protocols (Life Technologies).

#### hACE2 transduction

For transduction, WT or *Ifnar1*$^{-/-}$ mice were lightly anesthetized and instilled i.n. with $1x10^{11}$ DNase Resistant Gene Copies (DRGC) rAAV9-*eGFP* or rAAV9-*hACE2* in 100 µl PBS. *hACE2* gene expression in whole lung homogenate was assessed at day 20 post instillation by relative quantification to *Gapdh* using primers and probes for *hACE2* listed in the key resource table.

**Cryosectioning and native e*GFP* detection**

Mice were sacrificed 20 days post instillation of AAV-e*GFP* or PBS and lungs were removed after inflation with 4% PFA. After 24-hour fixation in 4% PFA, lungs were inflated with 30% sucrose and submerged in 30% sucrose for 24 hours. Lungs were subsequently inflated with 1:1 cryo embedding matrix (OCT)/30% sucrose and individual lobes were submerged in OCT/30% sucrose in plastic molds and frozen at -80 °C. Left lungs were cryosectioned to produce 7 µm thick sections, mounted using DAPI-supplemented mounting media with coverslip, and e*GFP* expression was detected by fluorescent microscopy using the EVOS FL Auto 2 system (Thermo Scientific).

**Virus and infections**

First wave SARS-CoV-2 (D614G, isolate of hCoV-19/England/IC19/2020) was grown in African green monkey kidney cells overexpressing human ACE2 and TMPRSS2 (Vero-ACE2-TMPRSS2; VAT cells) (22). For infection 20 days post transduction with rAAVs, mice were lightly anesthetized and instilled i.n. with $2x10^6$ plaque forming units (PFU) of SARS-CoV-2 in 100 µl volume. SARS-CoV-2 titre was assessed in lungs at 2, 4 and 8 d.p.i. using a plaque assay. In brief, serial dilutions of lung homogenate in serum-free Dulbecco's Modified Eagle Medium (DMEM, containing 1% non-essential amnio acids (NEAA), 100U/ml Penicillin and 100 µg/ml Streptomycin) were performed and inoculated onto VAT cells for 1 h at 37°C. The inoculum was then removed and replaced with overlay medium (1x MEM, 0.2% w/v BSA, 0.16% w/v $NaHCO_3$, 10 mM HEPES, 2 mM L-Glutamine, 100 U/ml penicillin, 100 µg/ml streptomycin and 0.84% agarose). Plates were incubated for 3 days at 37°C before overlay was removed and cells were stained for 1 h at room temperature in 2x crystal violet solution. Virus plaques were counted and multiplied by the dilution factor to calculate titer as PFU/ml.

**Isolation of lung cells**

Mice were sacrificed at 0.75, 2, 4 and 8 d.p.i. and lungs were perfused with PBS. To obtain lung leukocytes, lung lobes were cut into smaller pieces and incubated in complete DMEM (cDMEM, supplemented with 10% fetal bovine serum, 2mM L-glutamine, 100 U/ml penicillin and 100 µg/ml streptomycin), 1mg/ml Collagenase D (Roche) and 30 µg/ml DNase I (Invitrogen) for 1h at 37°C and then mashed through a 100-µm filter (BD). Red blood cells were lysed using Ammonium-Chloride-Potassium buffer.

**BAL cell processing**

BAL was collected by flushing the lungs three times with 1 ml PBS supplemented with 5 mM EDTA (Life Technologies). BAL cells and supernatant were separated by centrifugation and BAL supernatants

For Peer Review were exposed to UV light for 2 min to inactivate SARS-CoV-2. Red blood cells were lysed using Ammonium-Chloride-Potassium buffer.

**Flow cytometry**

After red blood cell lysis, lung and BAL cells were incubated for 30 min with fixable live-dead Aqua dye (Invitrogen), followed by fixation for 30 minutes with 4% paraformaldehyde (PFA) to inactivate virus. Cells were then incubated for 20 min with a purified rat $IgG_{2b}$ anti-mouse CD16/CD32 receptor antibody (BD) to block Fc binding, followed by staining with fluorochrome-conjugated antibodies against CD45 (30-F11, BV605), CD26 (H194-112, BV711), Siglec-F (E50-2440, BV786), Ly6G (1A8, AF488), Ly6C (12HK1.4, PE), CD11c (HL3, PE-CF594), CD64 (X54-5/7.1, APC) and CD11b (M1/70, AF700) in PBS containing 1% BSA and 5 mM EDTA for 25 min at 4°C. For the adaptive immune cells, they were stained with CD11c (HL3, V450), Siglec-F (E50-2440, BV786), CD19 (6D5, AF488), CD45 (30-F11, PerCP-Cy5.5), Ly6G (1A8, PE-Cy7) and CD3 (17A2, AF700). Samples were analysed on a BD-Fortessa Flow Cytometer equipped with 50-mW 504-nm, 50-mW 488-nm, 50-mW 561-nm and 20-mW 633-nm lasers and an ND1.0 filter in front of the FSC photodiode. All antibodies were purchased from BD, Biolegend or eBioscience. Data were analyzed with FlowJo software (Tree Star).

**RNA isolation and quantitative RT-PCR**

Lung tissue was homogenized in TRIzol and RNA extraction performed according to manufacturer's instructions. After the chloroform step, the aqueous phase containing RNA was further processed using the RNeasy Mini Kit (QIAGEN) according to manufacturer's instructions. 2 µg RNA was reverse transcribed using a High-Capacity RNA-to-cDNA kit (Applied Biosystems) according to manufacturer's instructions. To quantify mRNA levels in lung tissue, quantitative RT-PCR reactions for *Oas1, Viperin* and *Ifnl* were performed using primers and probes as previously described (23). Analysis was performed using the QuantiTect Probe PCR Master Mix (QIAGEN) and the 7500 Fast real-Time PCR System (Applied Biosystems). For absolute quantification, the exact number of copies of the gene of interest was calculated using a plasmid DNA standard curve, and the results were normalized to levels of *Gapdh* (Applied Biosystems). For relative quantification, the expression of *Cxcl1, Cxcl10, hACE2, Mx1* and SARS-CoV-2 *N* and *E* gene was expressed relatively to the expression of *Gapdh*. First, the ΔCT (CT = cycle threshold) between the target gene and *Gapdh* was calculated for each sample, followed by calculation of $2^{-\Delta CT}$. Analysis was performed using 7500 Fast System SDS Software (Applied Biosystems).

**Chemokine and IFN detection**

For Peer Review

CCL2 and IFN-λ2/3 quantifications were performed on BAL fluid using mouse DuoSet ELISA (R&D Systems) according to the manufacturer's instructions. Data were acquired on a SpectraMax Plus plate reader (Molecular Devices) and analysed using SoftMax software (version 5.2). The concentration of IFN-α and IFN-β was measured in BAL fluid using the Mouse ProCartaPlex Immunoassay (Invitrogen) according to the manufacturer's instructions. Data were acquired and analysed with a Bio-Plex 200 system (Bio-Rad Laboratories).

**Statistical analysis**

Statistical analysis was performed using Prism 9.2 (Graph-Pad Software). One-way ANOVA with Tukey's post-hoc test was used to compare multiple groups. Data are expressed as mean ± SEM, and for all tests a value of $P < 0.05$ was considered significant. * $P < 0.05$, ** $P < 0.01$, *** $P < 0.005$, **** $P < 0.001$

For Peer Review

### Acknowledgements

C. J. is supported by grants from UKRI-BBSRC (BB/V013831/1), Rosetrees Trust and Stoneygate Trust (M370 and M370-F1), Rosetrees Trust and The John Black Charitable Foundation (M956) and the Imperial College COVID-19 research fund. W.S.B., J.Z. and J.C.B are supported by the G2P-UK National Virology consortium funded by MRC/UKRI (grant ref: MR/W005611/1.). D.R.G., S.C.H. and Y.D are supported by a Wellcome Trust Portfolio grant (110579/Z/15/Z). For the purpose of open access, the authors have applied a CC BY public copyright license to any Author Accepted Manuscript version arising from this submission. The graphical abstract has been created using Biorender.com. We also thank the staff of St Mary's flow cytometry facility and the St Mary's animal facility for their assistance and members of the Johansson lab for scientific discussions.

### Author contribution

P.P.O. designed, performed, and analyzed the experiments and wrote the paper. M.G.M. and C.M. performed specific experiments and reviewed the paper. Y.D., D.R.G., S.C.H, O.H., and K.M.M. designed, manufactured and provided rAAV9-*hACE2* and rAAV9-*eGFP* and reviewed the paper, additionally Y.D. and K.M.M. performed specific experiments. J.Z., J.C.B. and W.S.B. provided advice and the SARS-CoV-2 WT isolate (D614G) and reviewed the paper. C.J. supervised the project, designed the experiments, and wrote the paper.

### Conflict of interest

The authors declare no commercial or financial conflict of interest.

### Data availability statement

The data that support the findings of this study are available from the corresponding author upon reasonable request.

### Ethics approval

All animal experiments were reviewed and approved by the Animal Welfare and Ethical Review Board (AWERB) at Imperial College London and approved by the UK Home Office in accordance with the Animals (Scientific Procedures) Act 1986 Amendment Regulations (PPL P3AFFF0DD Johansson).

For Peer Review

A) Recombinant Adeno-associated virus (rAAV) containing human angiotensin converting enzyme 2 (h*ACE2*) or *eGFP* genes was administered intranasally to *Ifnar1*$^{-/-}$ or wildtype (WT) mice (1x10$^{11}$ Dnase resistant gene copies/mouse). 20 days later mice were intranasally infected with SARS-CoV-2 (D614G, 2x10$^{6}$ PFU/mouse). Lungs and bronchoalveolar lavage (BAL) were harvested at 2, 4 and 8 days post infection (d.p.i). B) Expression of *hACE2* in lung tissue relative to *Gapdh*, measured by RT-PCR before infection (d20 post transduction with rAAV). WT/rAAV-e*GFP* n = 3, *Ifnar1*$^{-/-}$ /rAAV-e*GFP* n = 4, WT/rAAV-*hACE2* n = 4, *Ifnar1*$^{-/-}$ /rAAV-*hACE2* n = 5). C) Weight loss post infection with SARS-CoV-2. D) Viral load measured by plaque assay on Vero cells overexpressing hACE2 and TMPRESS2. E) Expression of SARS-CoV-2 *N gene* (nucleocapsid phosphoprotein) and *E gene* (envelope protein) in lung tissue relative to *Gapdh*, measured by RT-PCR. F) Gene expression analysis of IFN stimulated genes (ISG) *Cxcl10*, *Mx1*, *Oas1* and *Viperin* measured by RT-PCR, relative to expression of *Gapdh* or total copy numbers normalized to expression of *Gapdh (Oas1* and *Viperin).* Data are shown as mean ± SEM. B) WT/rAAV-e*GFP* n = 3, WT/rAAV-*hACE2* n = 4, *Ifnar1*$^{-/-}$ /rAAV-*hACE2* n = 5 C – F) Two independent experiments per time point, data pooled, n = 6-8 per group. One Way ANOVA + Tukey's multiple comparison test per time point; * indicates significant difference between WT rAAV-*hACE2* and *Ifnar1*$^{-/-}$ rAAV-*hACE2*, * P < 0.05, ** P < 0.01, *** p < 0.005, **** p < 0.001.

A) Gene expression of *Cxcl1* in lung tissue relative to *Gapdh* at 2, 4 and 8 d.p.i. with SARS-CoV-2 (D614G), measured by RT-PCR. B) Proportions of live, CD45$^{+}$ and total numbers of neutrophils in BAL at 2 d.p.i. C) Representative flow cytometry plots of lung cells gated on live, CD45$^{+}$ Ly6G$^{+}$ D) Proportions of live, CD45$^{+}$ and total numbers of neutrophils in lung tissue at 2, 4 and 8 d.p.i. Data are shown as mean ± SEM. Two independent experiments per time point, data pooled, n = 6-8 per group. One Way ANOVA + Tukey's multiple comparison test per time point; * indicates significant difference between *hACE2*-WT and *hACE2-Ifnar1*$^{-/-}$, * P < 0.05, ** P < 0.01, *** P < 0.005, **** P < 0.001.

A) Protein expression of CCL2 in BAL fluid at 2, 4 and 8 d.p.i. measured by ELISA. B) Proportions of live, CD45$^{+}$ and total numbers of CD64$^{+}$ CD11b$^{+}$ inflammatory myeloid cells in lung tissue at 2, 4 and 8 d.p.i. C) Representative flow cytometry plots of lung cells gated on live, CD45$^{+}$ Ly6G$^{-}$ SigF $^{-}$ CD11b$^{+}$. D) Proportions of live, CD45$^{+}$ and total numbers of CD64$^{+}$ CD11b$^{+}$ Ly6C$^{+}$ inflammatory myeloid cells in lung tissue at 2, 4 and 8 d.p.i. E) Proportions of live, CD45$^{+}$ and total numbers of CD64$^{+}$ CD11b$^{+}$ Ly6C$^{-}$ inflammatory myeloid cells in lung tissue at 2, 4, and 8 d.p.i. Data are shown as mean ± SEM. Two independent experiments per time point, data pooled, n = 6-8 per group. One Way ANOVA + Tukey's multiple comparison test per time point; * indicates significant difference between *hACE2*-WT and *hACE2-Ifnar1*$^{-/-}$, * P < 0.05, ** P < 0.01, *** P < 0.005, **** P < 0.001. Dotted line = limit of detection.

For Peer Review
